# Molecular Diagnostic Yield and Safety Profile of Ultrasound-Guided Lung Biopsies: A Cross-Sectional Study

**DOI:** 10.3390/cancers16162860

**Published:** 2024-08-16

**Authors:** Vito D’Agnano, Fabio Perrotta, Giulia Maria Stella, Raffaella Pagliaro, Filippo De Rosa, Francesco Saverio Cerqua, Angela Schiattarella, Edoardo Grella, Umberto Masi, Luigi Panico, Andrea Bianco, Carlo Iadevaia

**Affiliations:** 1 Department of Translational Medical Sciences, University of Campania L. Vanvitelli, 80131 Naples, Italy; fabio.perrotta@unicampania.it (F.P.); raffaella.pagliaro@studenti.unicampania.it (R.P.); angela.schiattarella1@studenti.unicampania.it (A.S.); edoardo.grella@unicampania.it (E.G.); umberto.masi@studenti.unicampania.it (U.M.); andrea.bianco@unicampania.it (A.B.); 2 U.O.C. Clinica Pneumologica L. Vanvitelli, Monaldi Hospital, A.O. dei Colli, 80131 Naples, Italy; fscerqua@gmail.com (F.S.C.); dott.carlo.iadevaia@gmail.com (C.I.); 3 Unit of Respiratory Diseases, Department of Medical Sciences and Infective Diseases, IRCCS Policlinico San Matteo Foundation, University of Pavia Medical School, 27100 Pavia, Italy; 4 Unit of Pathology Monaldi Hospital, A.O. dei Colli, 80131 Naples, Italy; filippo.derosa@ospedalideicolli.it (F.D.R.); luigi.panico@ospedaledeicolli.it (L.P.)

**Keywords:** lung cancer, ultrasound, biopsy, diagnosis, pathology, PD-L1, targeted therapy

## Abstract

**Simple Summary:**

An ultrasound-guided percutaneous lung biopsy performed by a pulmonologist is a safe, minimally invasive procedure for patients with suspected lung malignancies, providing an excellent diagnostic yield for a comprehensive molecular profiling and programmed death ligand 1 testing. Moreover, ultrasound-guided percutaneous lung biopsy may represent a successful approach for diagnosis of lung lymphoid lesions, with potential implication on reducing time-to-treatment time.

**Abstract:**

Background: The recent advances in precision oncology for lung cancer treatment has focused attention on the importance of obtaining appropriate specimens for tissue diagnosis as well as comprehensive molecular profiling. CT scan-guided biopsies and bronchoscopy are currently the main procedures employed for tissue sampling. However, growing evidence suggests that ultrasound-guided biopsies may represent an effective as well as safe approach in this diagnostic area. This study explores the safety and the diagnostic yield for cancer molecular profiling in ultrasound-guided percutaneous lung lesion biopsies (US-PLLB). Methods: One hundred consecutive patients with suspected lung cancer, between January 2021 and May 2024, who had ultrasound-guided lung biopsies have been retrospectively analyzed. Molecular profiling was conducted with next-generation sequencing Genexus using Oncomine precision assay or polymerase chain reaction according to specimen quality. Qualitative immunohistochemical assay of programmed death ligand 1 (PD-L1) expression was evaluated by the Dako PD-L1 immunohistochemistry 22C3 pharmDx assay. The co-primary endpoints were the molecular diagnostic yield and the safety profile of US-guided lung biopsies. Results: From January 2021 to May 2024, 100 US-guided lung biopsies were carried out and 95 were considered for inclusion in the study. US-PLLB provided informative tissue for a histological evaluation in 93 of 95 patients with an overall diagnostic accuracy of 96.84% [Sensitivity: 92.63%; Specificity: 96.84%; PPV: 100%; NPV: 100%]. Sixty-Six patients were diagnosed with NSCLC (69.47%) and were considered for molecular diagnostic yield evaluation and PD-L1 testing. Four patients had malignant lymphoid lesions. US-PLLB was not adequate to achieve a final diagnosis in three patients (3.16%). Complete molecular profiling and PD-L1 evaluation were achieved in all patients with adenocarcinoma (molecular diagnostic yield: 100%). PD-L1 evaluation was achieved in 28 of 29 patients (96.55%) with either SCC or NOS lung cancer. The overall complication rate was 9.47% (n = 9). Six patients (6.31%) developed pneumothorax, while three patients (3.16%) suffered mild haemoptysis without desaturation. Conclusions: According to our findings, US-guided lung biopsy is a safe, minimally invasive procedure in patients with suspected lung malignancies, providing an excellent diagnostic yield for both comprehensive molecular profiling and PD-L1 testing. In addition, our results suggest that US-guided biopsy may also be an effective diagnostic approach in patients with suspected lung lymphoma.

## 1. Introduction

Identification of genetic alterations amenable to therapeutic targeting and the approval of immune checkpoints inhibitors have led to an unprecedented expansion of the therapeutic landscape for non-small cell lung cancer (NSCLC) [1,2]. Considering the paradigm shift away from homogenous to a tailored therapeutic approach, histological distinction is still essential but no longer sufficient for making treatment decisions [3]. According to European Society for Medical Oncology, in patients with lung adenocarcinoma (LUAD) and in never or lighter smokers and younger patients with squamous cell carcinoma (SqCC), molecular biomarker profiling is mandatory to identify oncogenic drivers for which drugs are currently approved [4]. These include epidermal growth factor receptor (EGFR)—substitution, insertion, and deletion in exons 18-21—KRAS, HER2, and BRAF genes mutations; rearrangements involving the ALK, ROS1, NTRK1-3, and RET genes; and alteration in structure and/or expression of MET [4,5]. Likewise, membrane expression assessment of programmed death ligand 1 (PD-L1) through immunohistochemistry (IHC) represents a required selection criterion for the use of immunotherapy in both LUAD and SCC carcinoma. Specifically, patients with non-oncogene addicted, stage IV NSCLC—both SqCC and non-SqCC—are suitable for ICIs treatment, alone or in combination with chemotherapy, depending on whether PD-L1 expression assessed on at least 100 tumor cells (TCs) is greater than 50% or equal and/or greater than 1%, respectively [4]. In this scenario, the pivotal role of the respiratory physician is to optimize both bronchoscopic and transthoracic techniques to obtain samples suitable for complete molecular profiling [3,6]. Data on reliability of bronchoscopic procedures for genomic profiling have been published [7,8]. However, data regarding the diagnostic yield for genomic profiling for imaging-guided percutaneous lung biopsies remains scarce. In this study, we therefore sought to investigate the diagnostic yield for genomic profiling and evaluation of PD-L1 expression in ultrasound-guided percutaneous needle biopsy (US-PNB) of peripheral lung lesions, as well as its safety profile.

## 2. Materials and Methods

### 2.1. Study Design

This was a single centre retrospective study of US-guided PLLB in patients with suspected lung cancer. Informed consent was obtained from each patient prior to undergoing US-guided PLNB. The study was conducted in accordance with the Declaration of Helsinki. Ethical review and approval were not required due to the non-interventional retrospective study design.

### 2.2. Patients and Ultrasound Assessment of Lung Lesions

**Figure 1:**
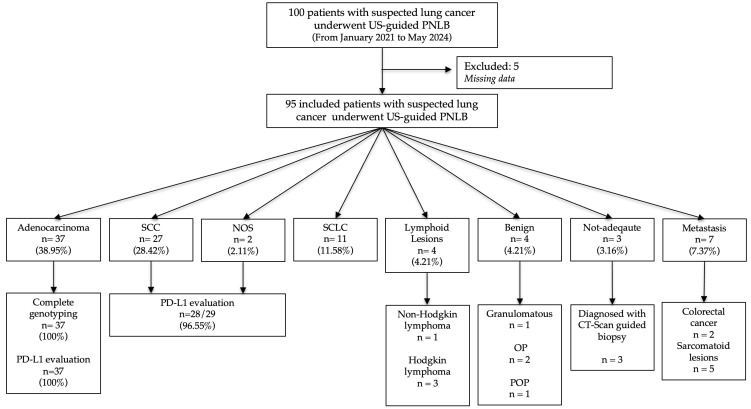
Patients’ flowchart. NOS: not otherwise specified; OP: organizing pneumonia; PD-L1: programmed cell death ligand 1; POP: post-obstructive pneumonia; SCC: squamous cell carcinoma; SCLC: small cell lung cancer; US-PNLB: ultrasound-guided percutaneous needle lung biopsy.

One hundred consecutive patients, admitted from January 2021 to May 2024 to the “Clinic of Respiratory Diseases L. Vanvitelli”, Monaldi Hospital, A.O. dei Colli, Naples Italy, with suspected lung cancer and for whom US-guided PLLB was required have been retrospectively analyzed and considered for study inclusion. Missing clinical data represented the exclusion criteria for this retrospective analysis evaluation. The patient flowchart is shown in Figure 1.

Demographic, clinical and radiological data from preprocedural contrast-enhanced total body CT and 18-FDG-PET-CT scan were recorded [9]. Consecutively, all patients underwent two-dimensional (B-mode) thoracic lung ultrasound. Anterior, lateral and posterior lung zones of each hemithorax were explored according to current guidelines [10]. Either curvilinear, convex, low frequency (3–7 MHz) or linear, high frequency (7–15 MHz) probe were used according to a patient’s habitus or lesion features. Color Doppler function was employed to confirm and exclude vascular structures. Patients with subpleural lung lesions with a diameter of more than 1 cm abutting the pleura and suspected of malignancy were considered suitable for US-PLNB.

Exclusion criteria for US-PNLB were severe respiratory failure, pneumothorax and uncontrolled cardiac arrythmia, and/or heart failure. Patients with rib fractures, altered mental status, severe anaemia, and with INR > 1.5 were not considered suitable for US-PNLB [11].

### 2.3. Intervention: US-Guided PLNB

All procedures were carried out under moderate sedation with intravenous midazolam (1–3 mg) and local anaesthesia using a 2% injectable lidocaine [8,9]. Procedures were performed in either a supine or prone position, depending on lesion localization. The US transducer was cleaned with povidone–iodine (Betadine^®^) (10% PVP-I) for at least 2 min to ensure sterilization. Likewise, skin disinfection was achieved using povidone–iodine (Betadine^®^) (10% PVP-I). Biopsies were carried out using either a 16- or 18-gauge semi-automatic spring-loaded biopsy system (Medax Velox 2^®^, Medax Srl, San Possidonio, Italy) under low frequency (3–7 MHz) or high frequency (7–15 MHz) ultrasound probe guidance with a free-hand technique. Needle size was selected according to the overall lesion dimension. One to three passages were performed according to lesion characteristics, patients’ tolerance, and quality of the specimens obtained. Tissue specimens obtained were expelled on a slide and immediately laid in embedding cassettes for biopsy, fixed in 10% formalin and sent for histopathological examination. After the procedure, the slides were stained with haematoxylin and eosin (H&E) in the pathologic laboratory and immediately analyzed by the pathologist and/or a trained respiratory physician for an immediate and preliminary evaluation of the specimen. Informed consent was obtained from each patient prior to undergoing US-PLLB.

### 2.4. Post-Intervention Management

After the procedure, sonography of the chest using linear probe was performed to exclude pneumothorax. Patients were followed-up for at least one hour post procedure depending on whether there were complications or not. Chest X-ray and a complete blood count test after 3 h were obtained. In cases of inadequate US-PLLB, cases were further discussed in lung cancer multidisciplinary teams regarding re-biopsy.

### 2.5. Assessment of Samples

**Figure 2:**
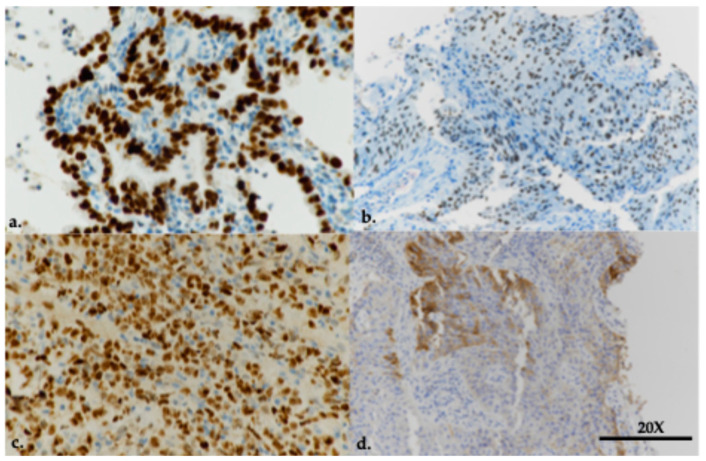
(**a**) Cell block obtained from ultrasound-guided percutaneous lung lesion biopsy (US-PLLB) demonstrating positivity thyroid transcription factor 1 (TTF-1): lung adenocarcinoma (LUAD); (**b**) Cell block from US-PLLB demonstrating positivity for p40 favouring the diagnosis of squamous cell carcinoma (SqCC); (**c**) cell block from US-PLLB showing positivity for B cell lymphoma 6 (BCL6): non-Hodgkin lymphoma; and (**d**) Dako programmed death ligand 1 (PD-L1) immunohistochemistry 22C3 assay showing an intermediate positivity expression for PD-L1 (range 1–49%).

Tissue specimens obtained were immediately laid in cassettes for cell block preparation and pre-fixed in 10% formalin. Successively, cell blocks were manually embedded in paraffin. Interpretation of specimens obtained with US-PLLB was performed by local pathologists, according to WHO guidelines [12]. Haematoxylin and eosin stain (H&E stain) was primarily used for morphological analysis. Immunostaining was also performed if samples were adequate. Positivity thyroid transcription factor 1 (TTF-1) antibodies (CONFIRM 8G7G3/1, Ventana, Roche, Tucson, AZ, USA) were considered consistent with the diagnosis of LUAD, since TTF-1 (Figure 2a) is positive in the majority of cases (70–80%), while positivity to p40 antibody (BC28, Ventana, Roche, Tucson, AZ, USA) (Figure 2b) and the negativity for TTF-1 favored the diagnosis of squamous cell carcinoma. Identification of neuroendocrine immunohistochemical markers—chromogranin (LK2H10, Ventana, Roche, Tucson, AZ, USA) and synaptophysin (CONFIRM SP11, Ventana, Roche, Tucson, AZ, USA)—was considered only in cases where neuroendocrine morphology was suspected. In case of clinical and/or morphology-based suspicion for lymphoid lung lesions, immunostaining was performed (Figure 2c). Evaluation of expression of PD-L1 was carried out using the Dako PD-L1 immunohistochemistry 22C3 pharmDx immunohistochemical assay (Dako, Glostrup, Denmark), where monoclonal antibodies (humanized IgG4) recognize the extracellular domain of PD-L1 to assess PD-L1 expression. The immunohistochemistry staining procedure was carried out on a Dako Autostainer Link 48 platform. PD-L1 expression was evaluated according to the tumor proportion score (TPS)—the percentage of viable tumor cells with at least partial membrane staining relative to all viable tumor cells in the examined section (Figure 2d). Molecular profiling was conducted with next-generation sequencing Genexus with Oncomine precision assay (Figure S1) or polymerase chain reaction according to the specimen quality.

### 2.6. Outcomes

The primary outcome was the molecular diagnostic yield of the US-guided PLLB. Specifically, this study aims to evaluate the success rate of complete cancer genotyping as well as the assessment of PD-L1 in cancers for which these analyses are required. The safety profile of US-guided PLLB represented a co-primary outcome of the present study.

### 2.7. Statistical Analysis

Demographic and clinical characteristics of the study population were summarized using mean, standard deviation, median, as well as counts and percentages, depending on their type and distribution. Sensitivity, specificity, positive predictive value (PPV), and negative predictive value (NPV) were calculated. Statistical differences between categorical variables were evaluated using chi-squared or Fisher’s exact test, as appropriate. Either a Student’s *t*-test or a Mann–Whitney test was considered to assess statistically significant differences for normally and non-normally distributed continuative variables, respectively. A two-tailed *p*-value less than 0.05 was considered statistically significant. All statistical calculations were performed with R software, version 4.4.0 (24 April 2024).

## 3. Results

Between January 2021 and May 2024, 100 patients with suspected lung cancer underwent US-guided PLLB. Five patients were excluded due to missing data. Therefore, 95 patients were included for retrospective analysis. The patients included had a median age of 68 years (range: 29–83). Characteristics of patients are summarized in the Table 1. US-PLNB provided informative tissue for a histological evaluation in 93 of 95 patients with an overall diagnostic accuracy of 96.84% [sensitivity: 92.63%; specificity: 96.84%; PPV: 100%; NPV: 100%].

**Figure 3:**
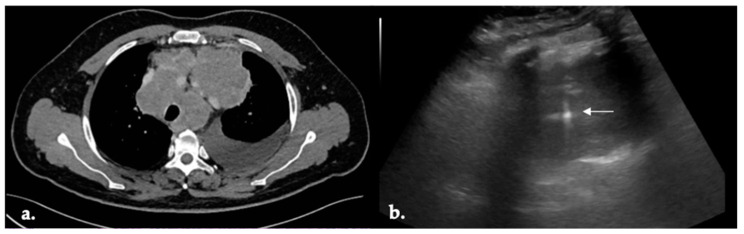
Ultrasound-guided percutaneous lung lesion biopsy (US-PLLB); Small cell lung cancer (SCLC). (**a**) Chest CT scan showing the target lesion; left pleural effusion was present; (**b**) out-of-plane 18 G US-PLLB; white arrow: needle.

**Figure 4:**
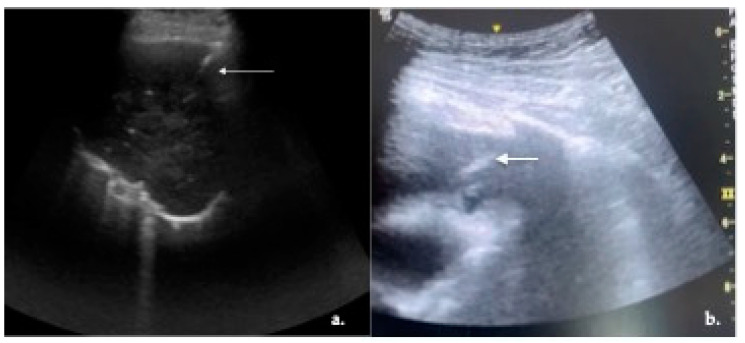
Ultrasound-guided percutaneous lung lesion biopsy (US-PLLB); (**a**) in-plane 18 G US-PLLB in patient with not otherwise specified non-small cell lung cancer (NOS-NSCLC); white arrow: needle; (**b**) in-plane 16 G US-PLLB in patients with non-Hodgkin lymphoma (NHL); white arrow: needle.

Sixty-six patients were diagnosed with NSCLC (69.47%) and were considered for molecular diagnostic yield evaluation and PD-L1 testing. Eleven patients (11.58%) had SCLC (Figure 3). Among the NSCLC patients, 37 (38.94%) had LUAD, 27 (28.42%) had SqCC, and 2 (2.11%) had not otherwise specified (NOS) NSCLC (Figure 4a). Three female patients (3.16%) had pulmonary involvement of Hodgkin lymphoma, while one patient (1.05%) had a lung involvement of non-Hodgkin lymphoma (Figure 4b). Among the seven patients (7.37%) who were diagnosed with lung metastasis, five of them (5.26%) had pulmonary involvement of metastatic sarcoma, while two (2.11%) had colorectal lung metastasis. Benign final diagnoses were lung granuloma, organizing pneumonia, and post-obstructive pneumonia. US-PLLB was not adequate for a final diagnosis in three patients (3.16%). After lung cancer multidisciplinary team (MDT) review, two patients underwent a CT scan-guided re-biopsy and one patient underwent an EBUS-TBNA. All patients were finally diagnosed with lung adenocarcinoma.

### 3.1. Molecular Diagnostic Yield and PD-L1 Expression Assessment

Complete molecular profiling and PD-L1 evaluation were achieved in all patients with adenocarcinoma (molecular diagnostic yield: 100%). KRAS mutation was detected in 11 patients (29.73%). The presence of EGFR mutation was detected in one patient (2.70%). Likewise, the presence of ALK mutation was detected in only one patient (2.70%). PD-L1 evaluation was achieved in 28 of 29 patients (96.55%) with either SCC or NOS lung cancer (Table 2). In our population, none amongst the available clinical characteristics were significantly associated with high PD-L1 expression (PD-L1 ≥ 50%) (Table 3).

### 3.2. Complications of US-Guided PLNB

The overall complication rate was 9.47% (n = 9). Six patients (6.31%) developed pneumothorax, while three patients (3.16%) suffered mild haemoptysis, without desaturation. All pneumothoraxes were promptly diagnosed by bedside chest ultrasound and confirmed with chest X-rays. All pneumothoraxes required chest drainage, performed by a respiratory physician. Patients were successively discharged within 3 to 5 days of hospitalization.

A total of six (9.33%) out of the seventy-five patients who were biopsied using an 18 G needle system experienced adverse events related to the procedures versus two (10%) out of the twenty patients in the group undergoing a 16 G US-PLLB. No significant difference in complication rates were observed between patients biopsied using either 18 G or 16 G [Odds ratio: 0.78; 95-CI: 0.12–8.58; *p* = 0.67] (Figure S2).

## 4. Discussion

According to The National Institute for Health and Care Excellence (NICE) guidelines, imaging-guided biopsy is currently recommended for patients with peripheral lung lesions when treatment can be planned on the basis of this test [13]. However, although most of the treatment decisions rely on tumor molecular profiling, a comprehensive genomic analysis remains inaccessible for a considerable proportion of patients leading to a reduction in therapy opportunities [14]. A physician’s skill in imaging-guided procedures, location of target lesions, and patients’ intolerance to the procedure may represent key obstacles to obtaining informative samples for both histological and molecular analysis [6]. Utilization of real time biopsy techniques, angling biopsy device to avoid central necrosis and a cytotechnologist verifying the presence of viable tumor cells in the specimen during ROSE have recently been proposed as potential solutions to address these issues [6,15]. Since the first studies exploring the safety, reliability and the successful rate of ultrasound-guided approach in the diagnosis of superficial lung lesions, advantages of this approach have been increasingly recognized. It represents a non-invasive, cost-effective, easy-to-learn technique that obtains real-time images of the target lesion avoiding normal lung and great vessels through the colour Doppler function. It has also been demonstrated to be a less time-consuming procedure when compared to CT scan-guided lung biopsies (29.5 ± 16.4 min vs. 37.6 ± 19.5 min, *p* = 0.007) and patients who underwent US-guided biopsy have significantly shorter waiting times compared to CT scan procedures [16].

In this retrospective study, we demonstrated that US-PLLB has an excellent overall diagnostic success rate, with a diagnostic yield of 97% (sensitivity: 96.84%; specificity: 100%; PPV: 100%; NPV: 100%). Our data are in accordance with data obtained by previous studies investigating the efficacy of US-guided transthoracic biopsies [12,17,18,19,20,21]. However, to our knowledge, this is the first study to investigate the molecular diagnostic yield for US-guided biopsy of lung lesions. We have demonstrated that US-PLNB has an excellent molecular diagnostic yield in patients with LUAD (diagnostic accuracy for molecular genotyping: 100%) and a diagnostic yield for PD-L1 evaluation of 100% and 96.3% in adenocarcinoma and squamous cell carcinoma, respectively. Data regarding the diagnostic yield for lung cancer genotyping have been reported in a cohort of patients undergoing US-guided biopsy of superficial lung cancer metastasis, including lymph nodes, subcutaneous, and pleural metastasis [22]. In this study, Livi and colleagues demonstrated an overall molecular diagnostic yield of 90.10%. Likewise, our study strongly suggests that US-guided procedures may have a key role in obtaining valid samples for both histological analysis and molecular profiling in patients with suspected lung cancer. Interestingly, the size of the cutting needle seems to be critical in terms of success rate [22]. As reported in both retrospective and prospective studies, a large-bore cutting needle provides more informative tissue in comparison to smaller bore needles. Pombesi and colleagues demonstrated that the rate of procedure-related complications is not significantly different between larger and smaller bore needle despite a higher diagnostic accuracy being observed in the former group (95.4% versus 85.8%, *p* = 0.0041) [17,22]. In our cohort, all procedures were carried out with large-bore—either 16 G or 18 G—semi-automatic spring-loaded biopsy system, without significant differences in terms of overall diagnostic accuracy between the two groups, despite all three patients requiring a re-biopsy in the 18 G group.

Regarding complication rate after the procedure, the most common adverse event was pneumothorax with an overall incidence rate of 6.31%, whilst three patients suffered mild haemoptysis, without desaturation, with an overall incidence rate of 3.16%. No significant differences with respect to complication rates have been observed between patients undergoing biopsy with either 18 G or 16 G needle, although the smaller number of 16 G procedures must be considered when analysing our results [6 (9.33%) vs. 2 (10.00%), 18 G versus 16 G, respectively; *p* = 0.67)]. Our results support the utilization of US-guided biopsy as method of choice for lung nodules abutting the pleura, as well as pleural-based lung lesions, in those centers with high expertise in US-guided procedures. Our results are in line with the literature data, confirming that pneumothorax and haemoptysis represent the main drawbacks of this approach, although incidence rate for pneumothorax after the procedure ranges from 3.3% to 12% [23,24]. Likewise, according to recently published data, haemoptysis and/or intraparenchymal haemorrhage rate after US-guided biopsy ranges from 1.3% to 8% [23,25,26]. These may partly be explained by different target lesions features, including diameter, peri-lesional emphysema or presence of air bronchogram, but also by operator experience [26].

Interestingly, we successfully diagnosed four cases of malignant lymphoid lesions by US-PLLB in our cohort, three cases of Hodgkin’s lymphoma (HL), and one case of non- Hodgkin lymphoma (NHL), respectively. Although primary lung NHL is rare, accounting for 0.4% of all lymphomas, pulmonary involvement of systemic NHL is reported in 4% to 25% of patients. Likewise, HLs are reported to involve lungs in a proportion of patients ranging from 15–40% [27,28]. Despite the small number of patients, our study suggests that US-guided biopsy may represent a successful technique (diagnostic accuracy: 100%) to obtain valid samples for a complete histopathologic characterization and classification, allowing prompt therapy initiation. This is of paramount importance considering that a longer time from diagnosis to treatment was demonstrated to be significantly associated with worse prognosis in patients with NHL [29].

Our results highlight the key role of the respiratory physician in the lung cancer diagnostic algorithm. To date, only the National Health Commission of the People’s Republic of China mentions ultrasound amongst recommended approaches for guided biopsy of both subpleural as well as metastatic lung cancer lesions [5,13,30]. Usage of ultrasound-guided biopsy should be encouraged in respiratory units for lesions considered suitable for this approach in light of its safety profile and high diagnostic accuracy.

We acknowledge limitations in our study. Retrospective and monocentric design represent two shortcomings. There is not a control group to compare outcomes with patients undergoing alternative procedures, especially CT scan-guided biopsies because all of our patients exhibited pleural-based lesions highly suspected to be malignant and suitable for an ultrasound approach. This made our cohort homogeneous for our study objective to evaluate molecular diagnostic yield from US-guided biopsies. To our knowledge, although some studies comparing ultrasound versus CT scan-guided subpleural lung lesions have shown no differences in terms of diagnostic accuracy [23], paucity of data remain regarding the method of choice in terms of molecular diagnostic yield in patients with NSCLC. Prospective, larger cohort studies are required to investigate this further. Furthermore, the procedures were carried out by clinicians who were skilled in this procedure, as well as by experienced pathologists. However, ultrasound-guided biopsies are easy-to-learn procedures that, in our opinion, should be supported by lung cancer multidisciplinary teams to improve diagnostic yield and reduce waiting times and costs.

## 5. Conclusions

Our results demonstrate that US-PLLB performed by respiratory physicians is a safe and effective procedure for obtaining samples for a comprehensive genomic analysis as well as for the PD-L1 expression testing in patients with NSCLC. In addition, our results suggest that US-guided needle biopsy may represent a successful approach for diagnosis of lung lymphoma. Larger multicenter studies are required to establish whether US-PLLB should be considered routinely for diagnosis of peripheral lung lesions.

## Figures and Tables

**Table 1:** Clinical characteristics of NSCLC patients according to the final diagnosis.

	Total	LUAD	SqCC	NOS
n (%)	66 (100%)	37 (56.06%)	27 (40.91%)	2 (3.03%)
Male (%)	53 (80%)	31 (83.78%)	20 (74.07%)	1 (50%)
Female (%)	13 (19.70%)	6 (16.22%)	7 (25.93%)	1 (50%)
Age (median)	69 (50–84)	68 (50–84)	69 (53–84)	70 (68–72)
<60 (%)	9 (13.64%)	6 (16.22%)	2 (7.41%)	/
60–74	40 (60.61%)	26 (70.27%)	13 (48.15%)	2 (100%)
>74	17 (25.76%)	5 (13.51%)	12 (44.44%)	/
Smoking (%)				
Current/Former	62 (93.94%)	35 (94.59%)	24 (88.89%)	2 (100%)
Never	4 (6.06%)	2 (5.41%)	3 (11.11%)	/
Gauge				
18 G (%)	55 (83.33%)	30 (81.08%)	23 (85.19%)	2 (100%)
16 G (%)	11 (16.67%)	7 (18.92%)	4 (14.81%)	/
Target Lobe				
RUL	22 (33.33%)	14 (37.84%)	6 (22.22%)	1 (50%)
RML	1 (1.52%)	1 (2.70%)	/	/
RLL	10 (15.15%)	5 (13.51%)	5 (18.52%)	1 (50%)
LUL	15 (22.73%)	6 (16.22%)	9 (33.33%)	/
LLL	18 (27.27%)	11 (29.73%)	7 (25.93%)	/
SUV mean (SD)				
Target Lesion	14.57 (5.66)	13.69 (5.63)	15.55 ((5.38)	17.85 (11.95)
Comorbidities				
Hypertension	32 (53.03%)	26 (70.27%)	5 (18.52%)	/
CAD	12 (18.18%)	7 (18.92%	5 (18.52%)	/
Dyslipidemia	28 (42.42%)	19 (51.35%)	8 (29.63%)	1 (50%)
Diabetes	24 (36.36%)	15 (40.54)	8 (29.63%)	1 (50%)
CKD	8 (12.12%)	2 (5.41%)	5 (18.52%)	1 (50%)
COPD	10 (15.15%)	7 (18.92%)	2 (7.41%)	1 (50%)
Dementia	3 (4.55%)	2 (5.41)	1 (3.70%)	/

CAD: coronary artery disease; CKD: chronic kidney disease; COPD: chronic obstructive pulmonary disease; NOS: not otherwise specified; LUAD: lung adenocarcinoma; LLL: left lower lobe; LUL: left upper lobe; RLL: right lower lobe; RML: right middle lobe; RUL: right upper lobe; SqCC: squamous cell carcinoma.

**Table 2:** Genomic profiling and PD-L1 expression assessment according to histology.

	Total	LUAD	SCC	NOS
EGFR (L858R Ex 21)	1	1	NA	NA
KRAS G12c	7	7	NA	NA
KRASG12X	4	4	NA	NA
ALK	1	1	NA	NA
PD-L1 (TPS)				
Total (n)	65/66	37/37	26/27	2/2
TPS < 1%	30	20	9	1
1%< TPS < 50%	19	8	10	1
TPS ≥ 50%	16	9	7	

PD-L1 expression assessment was achieved in all patients with LUAD and NOS lung cancer and in 26 of 27 patients with SCC. LUAD: lung adenocarcinoma; SCC: squamous cell carcinoma; NOS: not otherwise specified.

**Table 3:** Association between clinical characteristics and PD-L1 expression in NSCLC.

	TPS < 1%%	TPS ≥ 50%	*p*-Value
Total (n)	30	16	
Sex			
M	22 (73.33%)	13 (81.25%)	0.72 ^a^
F	8 (26.67%)	3 (18.75%)	
Age (median)	66.5	68	0.87 ^b^
IQR	11.5	8.25	
Smoking			
Yes	29 (96.67%)	14 (87.50%)	0.27 ^a^
No	1 (3.33%)	2 (12.50)	
SUVm (median)	15.17	13.42	0.612 ^b^
Target Lesion			
IQR	9.01	5.89	
Comorbidities			
Hypertension	20 (66.67%)	6 (37.50%)	0.11 ^c^
CAD	7 (23.33%)	3 (18.75%)	1.0 ^a^
Dyslipidaemia	14 (46.67%)	4 (25%)	0.20 ^c^
CKD	5 (16.67%)	1 (6.25%)	0.64 ^a^
COPD	6 (20%)	1 (6.25%)	0.39 ^a^

^a^: Fisher’s exact test; ^b^: Mann–Whitney U; ^c^: chi-square test. CAD: coronary artery disease; CKD: chronic kidney disease; COPD: chronic obstructive pulmonary disease.

## Data Availability

The data can be shared upon request.

## Supplementary Materials

The following supporting information can be downloaded at: https://www.mdpi.com/article/10.3390/cancers16162860/s1, Figure S1: Next-generation sequencing Genexus with Oncomine precision assay showed positive for KRAS mutation (G12X); Figure S2. US-PLLB: Complication rate by needle gauge in our population.
