# Supplementary material for: Molecular Diagnostic Yield and Safety Profile of Ultrasound-Guided Lung Biopsies: A Cross-Sectional Study
Source: Cancers (Basel). 2024 Aug 16;16(16):2860. doi: 10.3390/cancers16162860 (PMC11352358; doi:10.3390/cancers16162860)
Supplement: Supplementary file 1 [file cancers-16-02860-s001.zip › cancers-3124659-supplementary.pdf]

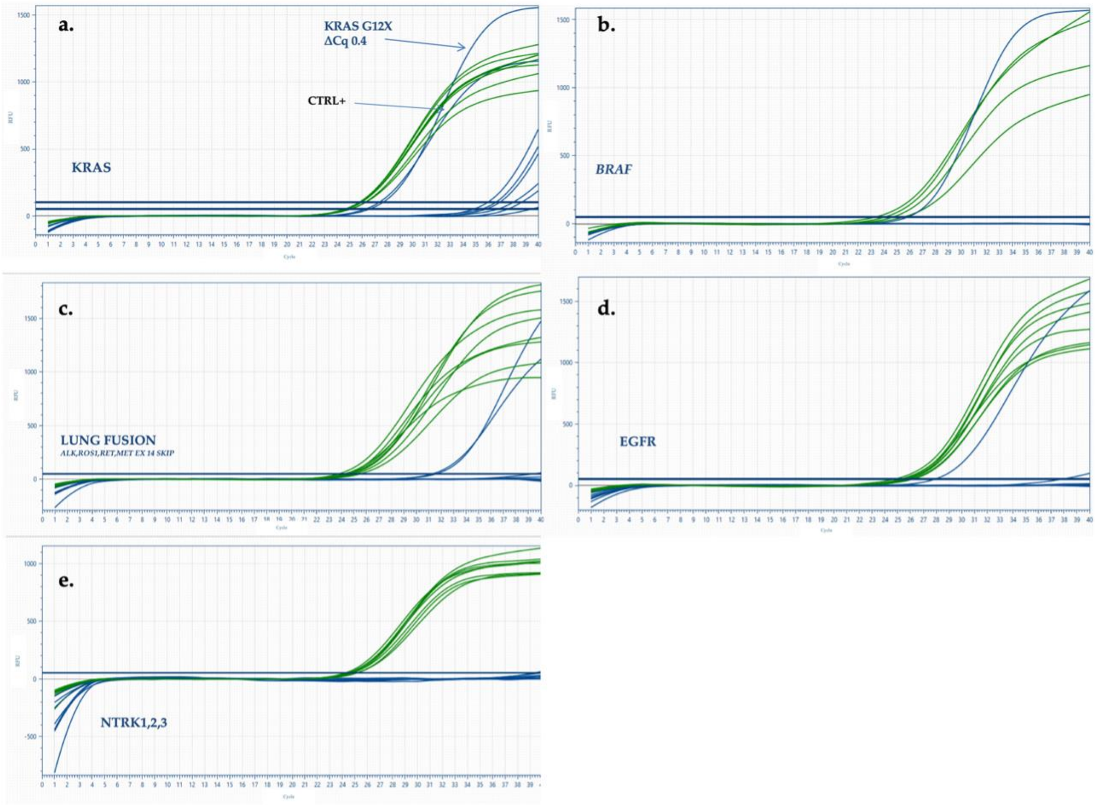

Figure S1. Next-generation sequencing Genexus with Oncomine precision assay showed positive for KRAS mutation (G12X).

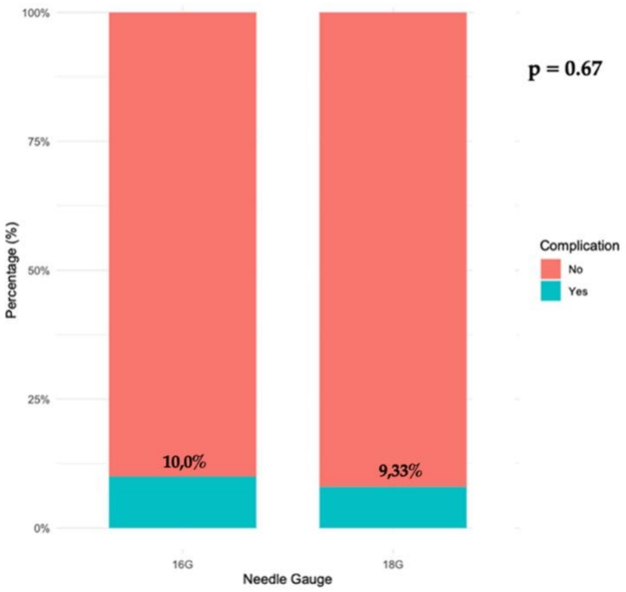

Figure S2. US-PLLB: Complication rate by needle gauge in our population
